# Innate immune checkpoint inhibitor resistance is associated with melanoma sub-types exhibiting invasive and de-differentiated gene expression signatures

**DOI:** 10.3389/fimmu.2022.955063

**Published:** 2022-09-28

**Authors:** Sultana Mehbuba Hossain, Gregory Gimenez, Peter A. Stockwell, Peter Tsai, Cristin G. Print, Janusz Rys, Bozena Cybulska-Stopa, Magda Ratajska, Agnieszka Harazin-Lechowska, Suzan Almomani, Christopher Jackson, Aniruddha Chatterjee, Michael R. Eccles

**Affiliations:** ^1^ Department of Pathology, Dunedin School of Medicine, University of Otago, Dunedin, New Zealand; ^2^ Maurice Wilkins Centre for Molecular Biodiscovery, Auckland, New Zealand; ^3^ Department of Molecular Medicine and Pathology, University of Auckland, Auckland, New Zealand; ^4^ Department of Clinical Oncology, Maria Sklodowska-Curie National Research Institute of Oncology, Krakow, Poland; ^5^ Department of Biology and Medical Genetics, Medical University of Gdansk, Gdansk, Poland; ^6^ Department of Medicine, Dunedin School of Medicine, University of Otago, Dunedin, New Zealand

**Keywords:** neoantigen, melanoma, immunotherapy, de-differentiation, neural crest-like, tumour mutation burden, cancer-associated fibroblast, gene expression signatures

## Abstract

Melanoma is a highly aggressive skin cancer, which, although highly immunogenic, frequently escapes the body’s immune defences. Immune checkpoint inhibitors (ICI), such as anti-PD1, anti-PDL1, and anti-CTLA4 antibodies lead to reactivation of immune pathways, promoting rejection of melanoma. However, the benefits of ICI therapy remain limited to a relatively small proportion of patients who do not exhibit ICI resistance. Moreover, the precise mechanisms underlying innate and acquired ICI resistance remain unclear. Here, we have investigated differences in melanoma tissues in responder and non-responder patients to anti-PD1 therapy in terms of tumour and immune cell gene-associated signatures. We performed multi-omics investigations on melanoma tumour tissues, which were collected from patients before starting treatment with anti-PD1 immune checkpoint inhibitors. Patients were subsequently categorized into responders and non-responders to anti-PD1 therapy based on RECIST criteria. Multi-omics analyses included RNA-Seq and NanoString analysis. From RNA-Seq data we carried out HLA phenotyping as well as gene enrichment analysis, pathway enrichment analysis and immune cell deconvolution studies. Consistent with previous studies, our data showed that responders to anti-PD1 therapy had higher immune scores (median immune score for responders = 0.1335, median immune score for non-responders = 0.05426, p-value = 0.01, Mann-Whitney U two-tailed exact test) compared to the non-responders. Responder melanomas were more highly enriched with a combination of CD8+ T cells, dendritic cells (p-value = 0.03) and an M1 subtype of macrophages (p-value = 0.001). In addition, melanomas from responder patients exhibited a more differentiated gene expression pattern, with high proliferative- and low invasive-associated gene expression signatures, whereas tumours from non-responders exhibited high invasive- and frequently neural crest-like cell type gene expression signatures. Our findings suggest that non-responder melanomas to anti-PD1 therapy exhibit a de-differentiated gene expression signature, associated with poorer immune cell infiltration, which establishes a gene expression pattern characteristic of innate resistance to anti-PD1 therapy. Improved understanding of tumour-intrinsic gene expression patterns associated with response to anti-PD1 therapy will help to identify predictive biomarkers of ICI response and may help to identify new targets for anticancer treatment, especially with a capacity to function as adjuvants to improve ICI outcomes.

## Introduction

The incidence of melanoma has increased continuously over the past 30 years (1) and cutaneous malignant melanoma accounts for 73% of skin cancer-related death (2). Although targeted therapies and immune checkpoint inhibitors (ICI) have become available to treat cutaneous melanoma (3), unfortunately, half of all the patients respond poorly to currently available targeted therapies and only 20–40% of melanoma patients respond well to anti-PD1 ICI monotherapies. Treatment with a combination of these drugs can result in approximately 60% response rates being achieved (4), but resistance to therapy and disease recurrence is very common, despite recent therapeutic advances provided by immunotherapy and targeted drugs. In addition, cancer ICI therapy has limitations, such as inability to accurately predict treatment efficacy and patient response. Furthermore, the therapy can lead to the development of cancer immunotherapy resistance, autoimmunity, and additionally there is overall high treatment cost (5).

Several studies have been conducted to investigate gene expression as a predictive biomarker for anti-PD1 monotherapy, or for anti-PD1 in combination with anti-CTLA4 monoclonal antibodies. Hugo et al. (6) conducted a study using 38 pre-treated (anti-PD1 therapy) cutaneous melanoma patient samples, and found that tumour mutation burden (TMB) was not sufficient to predict anti-PD1 therapy response. However overall high mutational loads were associated with improved survival. These authors also identified a transcriptional signature referred to as the IPRES, which indicates that non-responding melanomas up-regulate genes involved in mesenchymal transition, cell adhesion, extracellular matrix remodelling, angiogenesis, and wound healing. However, in this study the authors had used several samples from melanoma patients pre-treated with a MAPK inhibitor. The main limitations of using MAPK-inhibitor treated patients for ICI immunotherapy in melanoma is that the patients who relapse following MAPK pathway inhibitors, are frequently cross-resistant to immunotherapies too (7). In a later study, Riaz et al. (8) investigated 68 advanced melanoma samples to identify a biomarker for positive response to treatment with anti-PD1 monoclonal antibodies. They studied melanoma tissues before and after Nivolumab therapy and characterized activation of specific transcriptional networks, and upregulation of immune checkpoint genes. A limitation of this study was that they used different melanoma subtypes of melanoma patients, such as cutaneous, mucosal, uveal and other melanomas to identify response to anti-PD1 therapy. Unfortunately, patients with uveal melanoma do not receive benefit from anti-PD1 or anti-CTLA4 therapy, and the reasons behind poor ICI immunotherapy response in uveal melanoma are unclear (9). The action of immunotherapy usually relies on antigen-specific T cell responses by alleviating tumour-induced neoantigens (10). Cutaneous melanoma is a tumour type with one of the highest prevalence rates of somatic mutations. In contrast, uveal melanoma frequently presents with a low somatic mutation rate (11). The lack of neoantigens could be a possible reason for uveal melanoma not to respond to immunotherapy. For this reason, in our study we only used cutaneous melanoma samples to study the tumour microenvironment in response to anti-PD1 therapy.

The surrounding niche of melanoma, that is, the tumour microenvironment (TME), is composed of fibroblasts, endothelial cells, extracellular matrix (ECM), various immune cells, such as tumour infiltrating lymphocytes (TILs), as well as interactions with human leukocyte antigen class I antigens (HLA-1) expressed on tumour cells, and other tumour-associated factors, such as PD-L1, which together play a crucial role in melanoma development (12–15). Increased T cell infiltration and immunotherapy response is closely related to increased neoantigen [tumour-associated antigen (TAA) and tumour-specific antigen (TSA)] formation (16). Cancers frequently escape immune attack by promoting defective tumour antigen presentation, as well as secretion of immunosuppressive mediators, altering activated T-cells in both peripheral blood and lymph nodes and leading to the presence of inhibitory signals expressed by TILs in the TME (17, 18). Expression of PD-L1 protein is not the most accurate option for the prediction of response in melanoma (19), as patients with negative PD-L1 in immunohistochemistry can also frequently benefit from anti-PD-1 or anti-PD-L1 therapies (20, 21). Anti-PD1 therapy blocks interactions in the PD-1/L1 inhibitory axis and activates tumour-reactive T cells to induce anti-tumour responses (22, 23).

Primary (innate) resistance, and acquired resistance to ICI therapy, can occur in melanoma patients as a result of tumour immune escape mechanisms in antitumor immune response pathways regulated by the TME (24). Patterns of immune cell infiltration, changes in antitumor immune response pathways, or alterations of signalling pathways in tumour cells, and other changes in tumour cells can lead to an inhibitory immunosuppressive microenvironment and innate resistance (16). In contrast, the host immune system can facilitate tumour growth and progression by interactions between tumour cells, immune cells, and the TME, as an extrinsic factor (25).

In this study our goal was to gain a more comprehensive understanding of the immune infiltrate characteristics and malignant cell subsets in melanomas treated with ICI. Through an analysis of mRNA expression and TMB in cutaneous melanoma tissues between responding and non-responding patients, we have identified patterns of immune suppression, along with aggressive, invasive, and de-differentiated gene expression signatures, which we show correlate with poor response to ICI immunotherapy.

## Methods and materials

### Tumour specimens and profiling

From a Polish melanoma cohort, which was received from the Maria Sklodowska-Curie National Research Institute of Oncology, Krakow, Poland, a total of 40 patients’ samples were selected for the study. The patient selection criteria were – all the patients who received/are receiving Pembrolizumab or Nivolumab treatment (alone) for advanced stage 4 cutaneous melanoma, and who did not receive any previous cancer treatment, and where a sufficient amount of tissue was retrieved from the lymph node dissection (where available). The clinical data about the patients including the response to immunotherapy treatment, was collated from all melanoma patients. Ethical approval has been given for the Polish samples (KB/430-74/20).

### RNA and DNA extraction

We performed macrodissection to obtain cancer cells from heterogeneous histological samples. Hematoxylin and eosin (H&E) staining was performed to identify and mark the tumour regions on a representative slide-mounted tissue section for each tumour sample. The H&E-stained tumour slides were used as a reference for dissecting the area of interest from serial unstained slides from the same patients’ samples. Finally, a scalpel was used to carefully scrape off the tissues within the marked area of interest for downstream molecular analysis. RNA was extracted from macrodissected FFPE tissue samples using an Qiagen RNeasy mini prep kit, and then quantified using an Invitrogen™ Qubit™ RNA HS (High Sensitivity) Assay Kit. DNA was extracted using QIAamp DNA FFPE Tissue Kit, and quantified using an Invitrogen™ Qubit™ 1X dsDNA HS (High Sensitivity) Assay Kit. Isolated RNA and DNA concentrations were measured using an Invitrogen Qubit 4 fluorometer. Nanodrop assays were carried out to measure A260/A230 purity ratio to observe whether the isolation technique required further optimization.

### RNA sequencing and immune cell deconvolution

RNA integrity was determined using a 2100 Bioanalyzer system and associated RNA 6000 Nano kit (5067-1511; Agilent, CA, USA) to assess the quality of extracted RNA. Due to the low RNA yield and low RNA quality from approximately half the FFPE samples, we could only perform RNA-Seq analysis on 20 melanoma patient samples (responder = 10, non-responder = 10). For RNA-Seq, 500 ng of RNA for each sample was sent to the Otago Genomics Facility (OGF) where the RNA-Seq library preparation was performed using the ribo-depletion method (Gold). A TrueSeq mRNA stranded library prep kit (RS-122-2101; Illumina, CA, USA) was used for RNA library preparation. This was followed by running the RNA-Seq samples on an Illumina HiSeq 2500 sequencer (Illumina, USA) with single-end reads, read length of 101 basepairs and 20 million reads to produce raw fastq files.

The RNA-Seq reads were then adaptor trimmed using the cutadapt tool (26) and clean reads were then mapped to the human genome (assembly GRCh37) using HISAT2 (27). Quality base determination in the sequencing runs was performed using fastq-mcf tools, discarding results with a Phred score lower than Q20 (accuracy of 99%). The read counts for each sample were retrieved, first by exon, and then summarized by gene using featureCounts (28). To avoid spurious alignment, a mapping quality of 10 was used. Only high-quality data that had passed were further processed by using RStudio (Version 1.2.5042). The normalized gene expression levels were measured in TPM (Transcripts Per Million) values. Differential gene expression analysis using *edgeR* was performed to carry out CAMERA analysis. Further, normalised RNA-Seq data was used to estimate absolute abundance for immune and stromal cells in each sample using computational tools xCell package (29) and CIBERSORT (30).

### NanoString nCounter PanCancer Pathway Panel

Gene expression was measured using the NanoString nCounter PanCancer Pathways Panel (NanoString Technologies, Seattle, WA, USA). Each Panel consists of 770 genes, including 13 housekeeping genes. A total of eight tumour samples (four responder and four non-responder) from the same patients as were analysed by RNA-Seq, were subjected to NanoString analysis, to validate RNA-Seq gene expression data, and to perform cancer pathway analysis. The samples were selected based on mapping efficiency (more than 90%) to the human genome (assembly GRCh37) based on our RNA-Seq data analysis. For each NanoString assay, 1 μg of total tissue RNA was isolated, mixed with a NanoString code set mix and incubated at 65°C overnight (16–18 hr). The reaction mixes were loaded on the NanoString nCounter Prep Station for binding and washing, and the resulting cartridge was transferred to the NanoString nCounter digital analyzer for scanning and data collection. Raw count data was preprocessed using the geNorm algorithm in nCounter Advanced Analysis ver. 2.0.115 (NanoString Technologies) (31). A quality check of raw data was conducted using nSolver Analysis Software ver. 4.0 and NanoStringQCpro ver. 1.14.0 (NanoString Technologies). Z-scores were generated for each gene from normalised data to perform further analysis.

### Tumour mutation burden panel analysis

DNA quantification for Ion AmpliSeq™ Library Preparation was carried out using TaqMan^®^ RNase P Detection Reagents, as described by the manufacturer (ThermoFisher). DNA samples were first quantitated, and the DNAs were then shipped for analysis using Oncomine™ Tumor Mutation Load Assays, which were carried out as described by the manufacturer (ThermoFisher, Melbourne, Australia).

### Bioinformatics methods used to analyse TMB data

The data were received in a BAM file format to process for further analysis. The initial data was analysed on Oncomine Tumour Mutation Load – w3.0 – DNA – Single Sample in the Ion Reporter cloud server. We selected only 31 samples for further analysis because these samples passed in-built QC (quality control) tests with ≥80% depth reads uniformity in the whole genome. Then the mutation load, driver mutation, nsSNVs, mutation type and mutation location were identified for each sample (p-value < 0.05). Then the multiple genomic alteration events were presented by OncoPrint heatmap using RStudio.

### Neoantigen analysis

We used pVAC-tools 3.0.0 (*pVAC-Seq* command) pipeline to predict neoantigen production from each sample of RNA sequencing data. We used an RNA variant-calling pipeline to identify a list of somatic non-synonymous mutations and then annotated transcripts sequence for changed amino acids, followed by *in silico* approach to determine HLA haplotypes of the patient. At first, the fastq files from RNA sequencing were trimmed using Cutadapt3.4 and aligned using STAR 2.2.2.5 against the human genome (assembly GRCh37). We performed local indel rearrangement, base quality score recalibration and RNA variant calling using GATK 2.2.0 with default parameters (32, 33). Variants with known dbsnp138 ID were excluded from subsequent neoantigen analysis as those are likely to be germline. In parallel, RNA-Seq data were analysed using OptiType v1.3 to predict the MHC class I alleles haplotype of each patient. Kallisto 0.46.2 (GRCh37, genecode v19) was used to quantify abundances of transcripts and genes from the RNA-Seq data. To predict high affinity peptides that are likely to bind to the MHC class I molecule, we used four different epitope prediction softwares: MHCflurry, NetMHC, NetMHCpan and PickPocket. To streamline the comparison, we first built an output file that consisted of two amino acid sequences per variant site: wildtype WT (normal) and mutant MT (tumour). Then the best MT score was calculated based on the lowest binding score for MT sequence with the localized peptides and per mutation between all independent HLA alleles that were used as input.

### Bioinformatics methods used to analyse online available data

For validation purposes, we used publicly available melanoma data accessed through the Gene Expression Omnibus (GEO, URL: https://www.ncbi.nlm.nih.gov/geo/) from NCBI, associated with recent publications (6, 8). These data included RNA-Seq data for pre-treated melanoma patients using anti-PD1 therapy, and were downloaded as SRA files (raw sequence data), followed by using *fastq-dump* to produce fastq files. After that, we used an in-house pipeline to generate read counts for each sample. To maintain the consistency with our patients’ categorization, we excluded the samples which were pre-treated with MAPK-inhibitors from the GSE78220 dataset, and only used cutaneous pre-treated melanoma samples from the GSE91061 dataset.

### Statistical analysis

Gene Set Enrichment Analysis (GSEA) was performed using gene sets available in the Broad Institute Molecular Signatures Database (MsigDB) which included H1 (hallmark), C2 (curated), and C5 (gene ontology) (34). CAMERA tests were performed as available from the *edgeR* package and a FDR adjusted p value ≤ 0.05 used as the statistically significant threshold (35). For generating a gene-set score for each sample, single sample GSEA (ssGSEA) (36) was used from the GSVA Bioconductor R package (37). The endothelial-mesenchymal transition (EMT) score, viral mimicry score, and differentiation score were self-curated from different published articles. The viral mimicry gene sets, which included *DDX58*, *DDX41*, *IFIH1*, *OASL*, *IRF7*, *IRF1*, *ISG15*, *MAVS*, *IFI27*, *IFI44*, *IFI44L*, and *IFI16*, were obtained from our previously published work (38). EMT signature genes including *CDH1*, *EPCAM*, *GRHL2*, *KRT19*, *RAB25*, *CDH2*, *VIM*, *ZEB1*, *ZEB2*, *SNAI2* and *TWIST1* were collected from Tan and colleagues (39) and also merged with “HALLMARK_EPITHELIAL_MESENCHYMAL_TRANSITION” from MsigDB. Proliferative signature genes that were used were from the Widmer et al. identified dataset (40). Differentiation signature genes (see **Supplementary Table S1**) from Tsoi and colleagues (41) were acquired to further evaluate the dedifferentiation signature in our patients’ derived samples. These genes included 7 groups, in order, from least differentiated to the most differentiated: (1) Undifferentiated, (2) Undifferentiated-Neural crest-like, (3) Neural crest-like, (4) Neural crest-like-Transitory (5) Transitory, (6) Transitory-Melanocytic, (7) Melanocytic. Z-scores were generated for each gene and unsupervised hierarchical clustering was performed. Mann-Whitney U test was performed using GraphPad Prism software (Version 8, GraphPad Software, Inc., San Diego, CA, USA).

## Results

### Patients’ characteristics

In this investigation, formalin-fixed paraffin-embedded (FFPE) melanoma tissue samples were obtained from 40 melanoma patients, who had been treated with anti-PD-1 monotherapy (nivolumab or pembrolizumab). The patients did not receive any prior treatment, and all FFPE tissues were collected before treatment was started. The tissues were obtained from either primary melanoma or lymph node metastases, which had been resected prior to starting immunotherapy treatment for diagnostic purposes. Responders were defined as patients with a RECIST 1.1 criteria (42) – complete response (CR), partial response (PR), or stable disease (SD) of greater than 6 months with no progression. Non-responders were defined as progressive disease (PD) or SD for less than or equal to 6 months before disease progression. We stratified our dataset into 19 responder and 21 non-responders (**Table 1**).

**Table 1:** Clinicopathological features of the melanoma patients (refer to **Supplementary Text**).

	Total	BRAF^V600E^Mutation	Responder for the ICI (RECIST criteria)
	n = 40	n = 9	n = 19
Age (years)*			
Median = 65			
Range = 34 – 90			
<40	2	1	1
40 – 60	11	5	5
>60	27	3	13
Gender			
Male	21	6	11
Female	19	3	8
No patients who have positive LNs	25	8	13

*Age is the year of surgery.

### Responder melanomas exhibit higher mutation burden compared to non-responder melanomas

**Figure 1:**
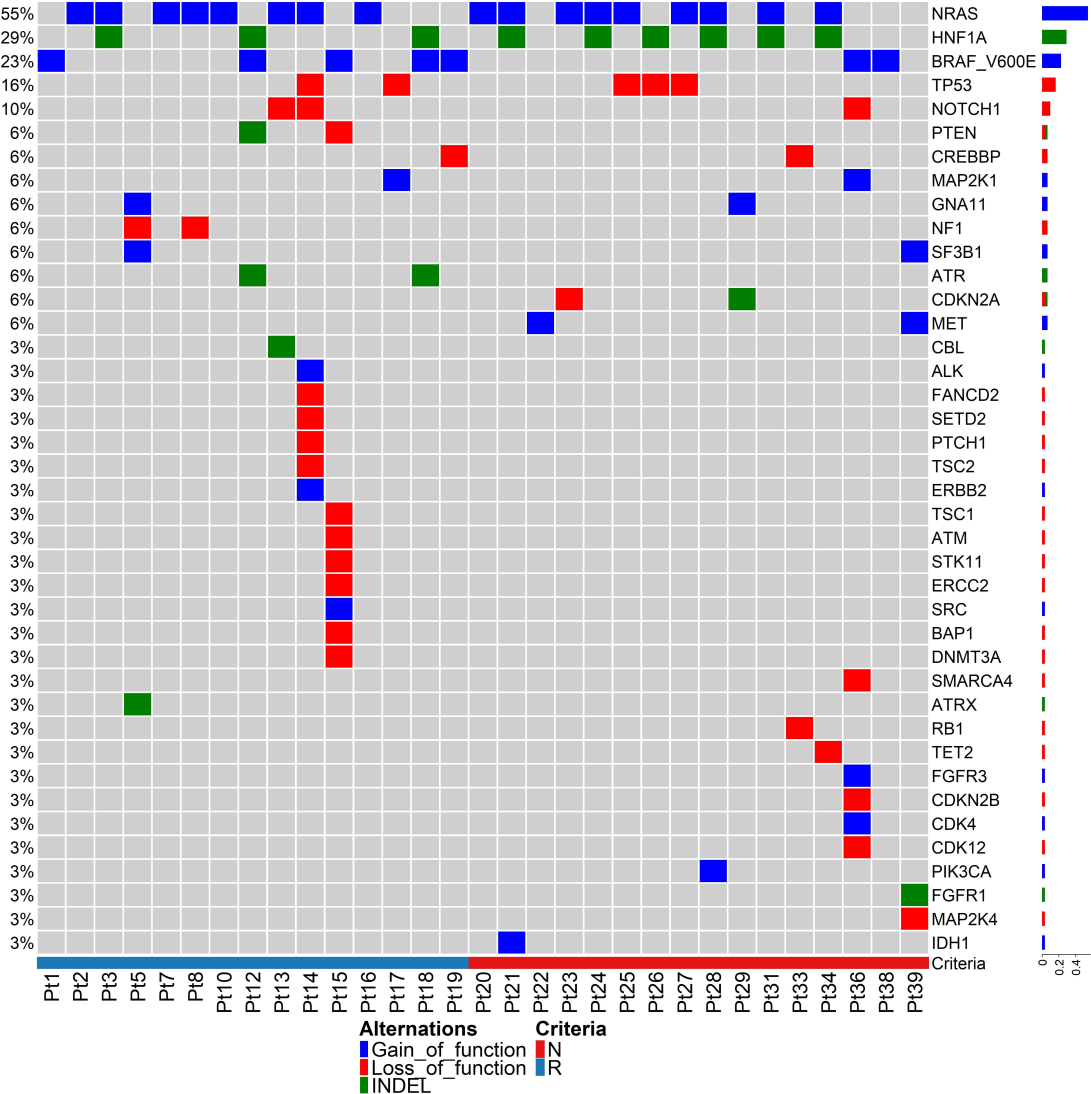
Tumour mutation analysis between responding and non-responding melanomas. Driver mutations in pre-treatment melanomas of responding versus non-responding patients prior to anti-PD-1 therapy. The heatmap represents only 31 samples because these samples passed an in-built QC (quality control) test with ≥80% depth of read uniformity in sequencing using the Oncomine Tumour Mutation Load – w3.0 – DNA – Single Sample in the Ion Reporter cloud server.

Clonal mutations were more frequent in responders than non-responders, with a median TMB value of 16.7 and 8.5, respectively (p = 0.07). *NRAS*, *HNF1A*, *BRAF^V600E^*, *TP53* and *NOTCH1* were the most common driver mutations in this patient cohort (**Figure 1**), with *NRAS* gain of function mutations being the most common (n=17/31 patients; 55%). *BRAF^V600E^* mutations were identified in 23% cases (n=9/40). These results are consistent with previous studies (6). No melanomas were observed with both *NRAS* and *BRAF^V600E^* mutations. Mutation in *NRAS* causes reactivation of the MAPK pathway, and corresponding *NRAS* and *BRAF* mutations in single cells are very rare due to self-induced apoptosis by sustained hyper-activation of the MAPK pathway (43). An indel mutation in *HNF1A* was identified in 29% of cases, and gain of function mutations in the *MET* oncogene were observed in 2 non-responding melanomas. Mutations in *HNF1A* (Hepatocyte Nuclear Factor 1A) have previously been identified in hepatoma, colon cancer and endometrial cancer, and *HNF1A* gene mutations are associated with risk of pancreatic cancer (44).

Our data indicate that most of the driver mutant genes are involved in DNA damage repair pathways (**Figure 1**), such as *TP53*, *NOTCH1*, *PTEN*, *CREBBP*, *MAP2K1*, *SF3B1*, *MET*, *ATM*, *ALK*, *SETD2*, *ERCC2*, *BAP1*, *DNMT3A*, *SMARCA4*, *ATRX*, *RB1*, *CDK12*, and *IDH1*, indicating genomic instability in melanoma. There were no mutations in specific genes that strongly correlated with response or resistance to anti-PD1. The *ZNF384* gene harboured nsSNVs in 8 of 16 non-responding tumours (50%), but in only 2 of 15 responding tumours (13.3%) (**Supplementary Figure S1**).

### Relatively lower immune scores occur in non-responding melanomas

**Figure 2:**
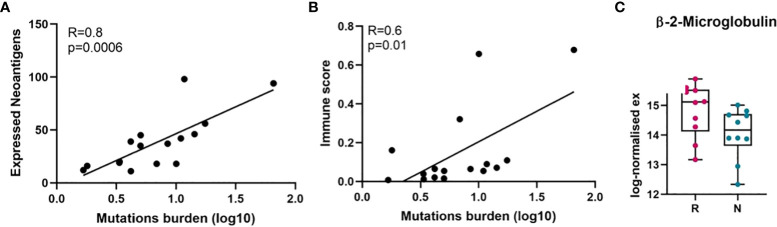
Correlation of mutation burden with neoantigens and immune score analysis. **(A)** Pearson correlation between tumour mutation burden and expressed neoantigens in melanomas. **(B)** Pearson correlation between tumour mutation burden and immune score in melanomas. **(C)** Beta-2-microglobulin (*B2M*) expression level in responder and non-responder tumours in log-normalised expression value (p = 0.06, not significant, Mann-Whitney U). Here, because RNA-Seq was performed on twenty samples, it was only possible to match twenty samples from TMB and RNA-Seq data (responder = 10, non-responder = 10) were used to perform Pearson correlation analysis. On the horizontal axis in C), R, responder; N, non-responder.

To better understand the immune landscape of melanoma, neoantigens from the FFPE tumour samples in our cohort were predicted by using the pVAC-tools bioinformatics pipeline on our RNA-Seq raw data files. In total, we found 5457 neoantigens predicted to have a high binding affinity to human leukocyte antigen I (HLA-I) (IC50 < 500 nanomolar (nM)), and a lower HLA-I binding affinity (IC50 > 500 nM) to the corresponding wildtype peptides. Since immune cells recognize neoantigens that are expressed and presented by HLA molecules on the tumour cell surface, we filtered out the 788 best binding epitopes, which passed the above criteria, and had strong MT binding (IC50 < 500) and strong expression (TPM * RNA_VAF > 3). While the predicted neoantigens were directly derived from somatic mutations and were expected to correlate with TMB, we confirmed that the expressed neoantigens had a statistically significant correlation with TMB in all samples (Pearson correlation, R = 0.8, p = 0.0006, two-tailed) (**Figure 2A**).

Moreover, it is known that cancer cells with high tumour antigen loads may avoid presenting neoantigens on the surface of MHC, through altering the antigen presenting machinery such as proteasome subunits or transporters associated with antigen processing (TAP), beta-2-microglobulin (*B2M*) or MHC itself (45, 46). Therefore, we investigated the expression level of beta-2-microglobulin mRNA (*B2M*) and human leukocyte antigen (HLA) in both groups of patients and found the expression of *B2M* is downregulated compared to the responding tumours (**Figure 2C**, **Supplementary Figure S2**). Downregulation of *B2M* and HLA was observed in non-responder melanomas, although these were not significant.

Given the overall strong correlation between TMB and the expressed neoantigens, we next examined the correlation between TMB and tumour immune score. In our cohort, TMB showed positive correlation with tumour immune score (Pearson correlation, R = 0.6, p = 0.01, two-tailed) (**Figure 2B**), suggesting that higher TMB may cause increased expression of neoantigens leading to activation of immune response.

### The non-responding tumour microenvironment promotes immunosuppression, and enhanced aggressiveness and malignancy

**Figure 3:**
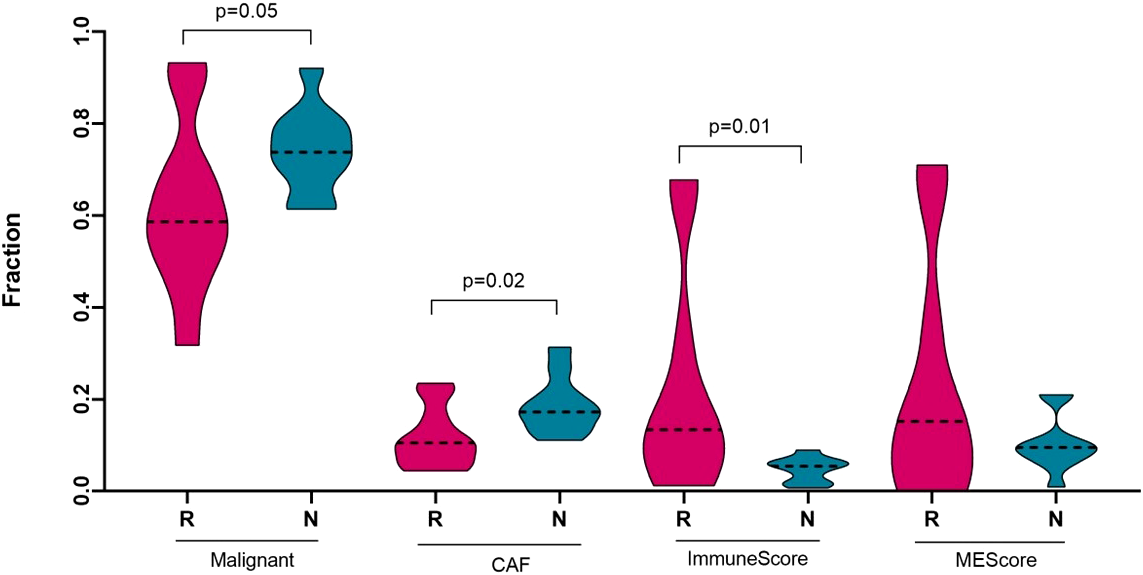
Deconvolution study for malignant cells, cancer associated fibroblasts and immune score between responding (n = 10) and non-responding (n = 10) melanomas. Significance tests were performed using Mann–Whitney U test. On the horizontal axis, R, responder; N, non-responder; CAF, cancer associated fibroblast; and MEScore, micro-environment score.

High TMB (non-synonymous mutations) is a tumour-intrinsic feature and is associated with antitumor immune responses and responses to ICIs (47, 48). To determine the tumour microenvironment of cell subsets in each patient’s sample and to compare between responding and non-responding melanomas, we performed CIBERSORT and xCell analyses. Gene expression values in transcripts per million (TPM) were used to estimate the immune cell content, and to calculate an immune score by analysis using the xCell package. [54]. The immune score was calculated based on quantifying the density and location of immune cells within the tumour. The median immune scores for responders and non-responders were 0.1335 and 0.05426, respectively, with a p-value of 0.01 Mann-Whitney U two-tailed exact test) (**Figure 3**), suggesting that high numbers of TILs are present in responders to anti-PD1 therapy, and which is indicative that responding tumours are highly antigenic and melanocytic in nature, while in contrast non-responding tumours are metastatic in nature.

To characterize the tumour microenvironment of responder and non-responder groups of melanomas, we performed CIBERSORTx, which is an *in silico* algorithm (49) for estimating specific cell types in a mixed cell population, using transcriptomic data, which were available for 20 melanoma patients from our cohort. A reference marker gene or signature matrix was prepared using the Tirosh et al. (50) single cell RNA-Seq metastatic melanoma dataset. This signature matrix was used to acquire the abundance of melanoma malignant cells and cancer associated fibroblast (CAFs) cell subsets in each sample. Non-responding melanomas were highly enriched with malignant cell types (p-value = 0.05) and with CAFs cell subsets (p-value = 0.02) compared to the responding melanomas (**Figure 3**). Previously, several studies reported that melanoma-associated fibroblasts can induce immune suppression *via* melanoma–stroma crosstalk (51–53), which prompted us to check the immune cell component in the tumour microenvironment of the responders and non-responders to the anti-PD1 therapy.

**Figure 4:**
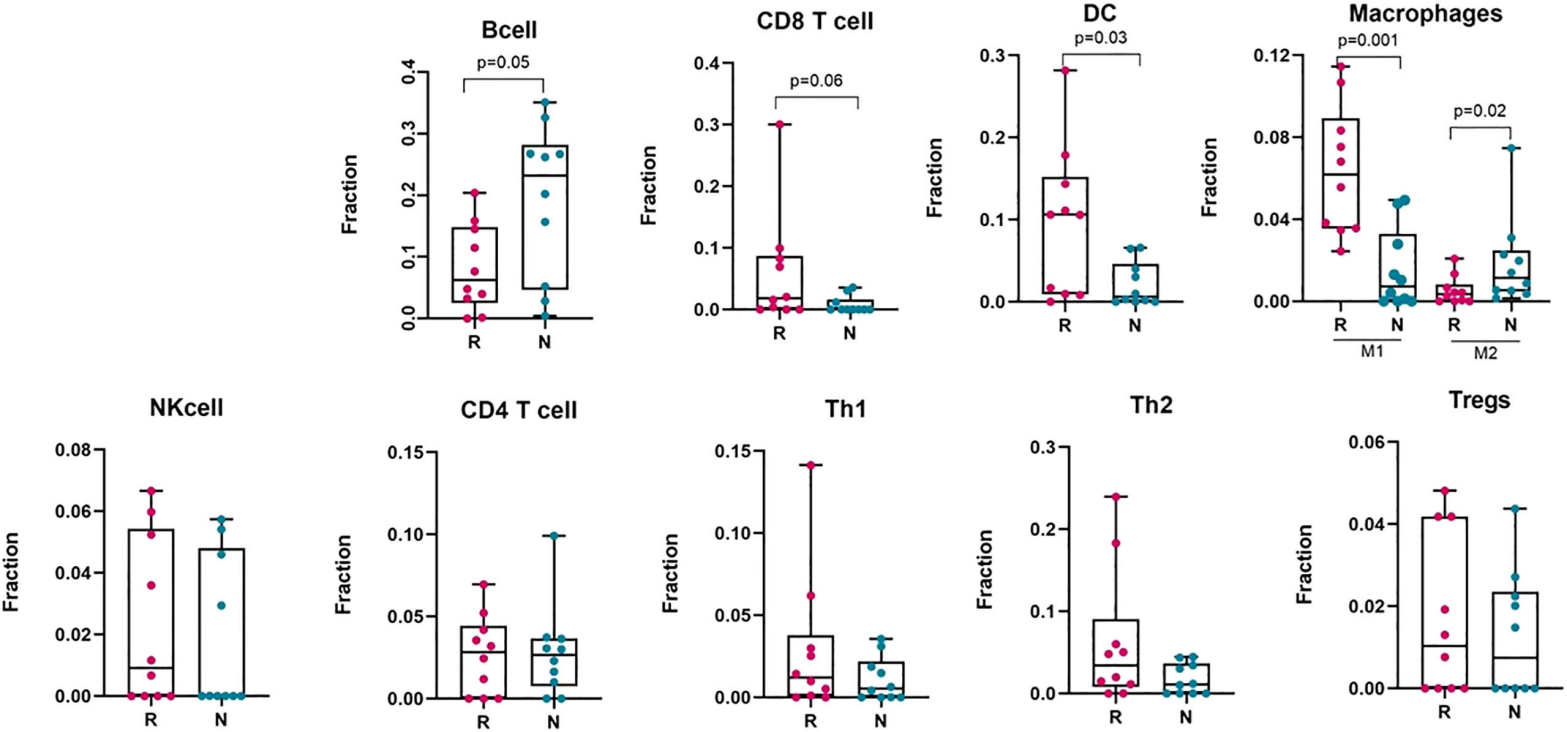
Deconvolution study to estimate different immune cell abundance in responding (n = 10) and non-responding (n = 10) melanoma tissues. Significance tests were performed using Mann–Whitney U test. Here, R, responder; N, non-responder, DC, dendritic cells; NK cell, Natural killer cell; Th1, T helper cell 1; Th2, T helper cell 2; Tregs, regulatory T cells.

The tumour microenvironment of melanoma contains many immune cells or tumour infiltrating lymphocytes (TILs), such as different subsets of T−cells, dendritic cells, macrophages, neutrophils, mast cells, B lymphocytes, and natural killer (NK) cells (54). To estimate the presence of different immune subtypes in the TME, we used a microarray derived LM22 signature matrix (55) for profiling 22 functionally defined human immune cell types in each sample. Responder melanomas were more highly enriched with a combination of CD8+ T cells, T helper cells (Th1 and Th2), dendritic cells (p-value = 0.03) and an M1 subtype of macrophages (p-value = 0.001) (**Figure 4**). Interestingly, non-responding tumours were replete with an M2 subtype of macrophages (p-value = 0.02) and B-cells (p = 0.05). We identified that non-responding tumours were significantly enriched with CAFs, M2 macrophages, malignant cell types and lower immune score, which we hypothesize promotes immunosuppression, tumour aggressiveness and distant metastasis of this group of melanomas.

To validate the immune cell proportions in responding and non-responding melanomas, we performed an analysis with the CIBERSORT and xCell packages on pre-treated melanoma samples, using bulk RNA-Seq datasets (GEO: GSE78220) and (GEO: GSE91061) (**Supplementary Figures S3A, B**). To categorize the samples as responder and non-responder, we followed precisely the information provided with the GSE78220 dataset, and for the GSE91061 dataset, the patients with complete response (CR) and partial response (PR) were defined as responders, and stable disease (SD) and progressive disease (PD) were defined as non-responders following the RECIST 1.1 criteria. These analyses gave similar patterns as our own dataset for the presence of immune cells, such as dendritic cells, M1 and M2 subtypes of macrophages, CD8+ T cells, CD4+ T cells and naïve B cells in the melanoma tumour bed, which can discriminate between responders and non-responders to anti-PD1 therapy.

### Non-responding melanomas to immunotherapy represent a state of de-differentiation and neural crest-like gene signatures

**Figure 5:**
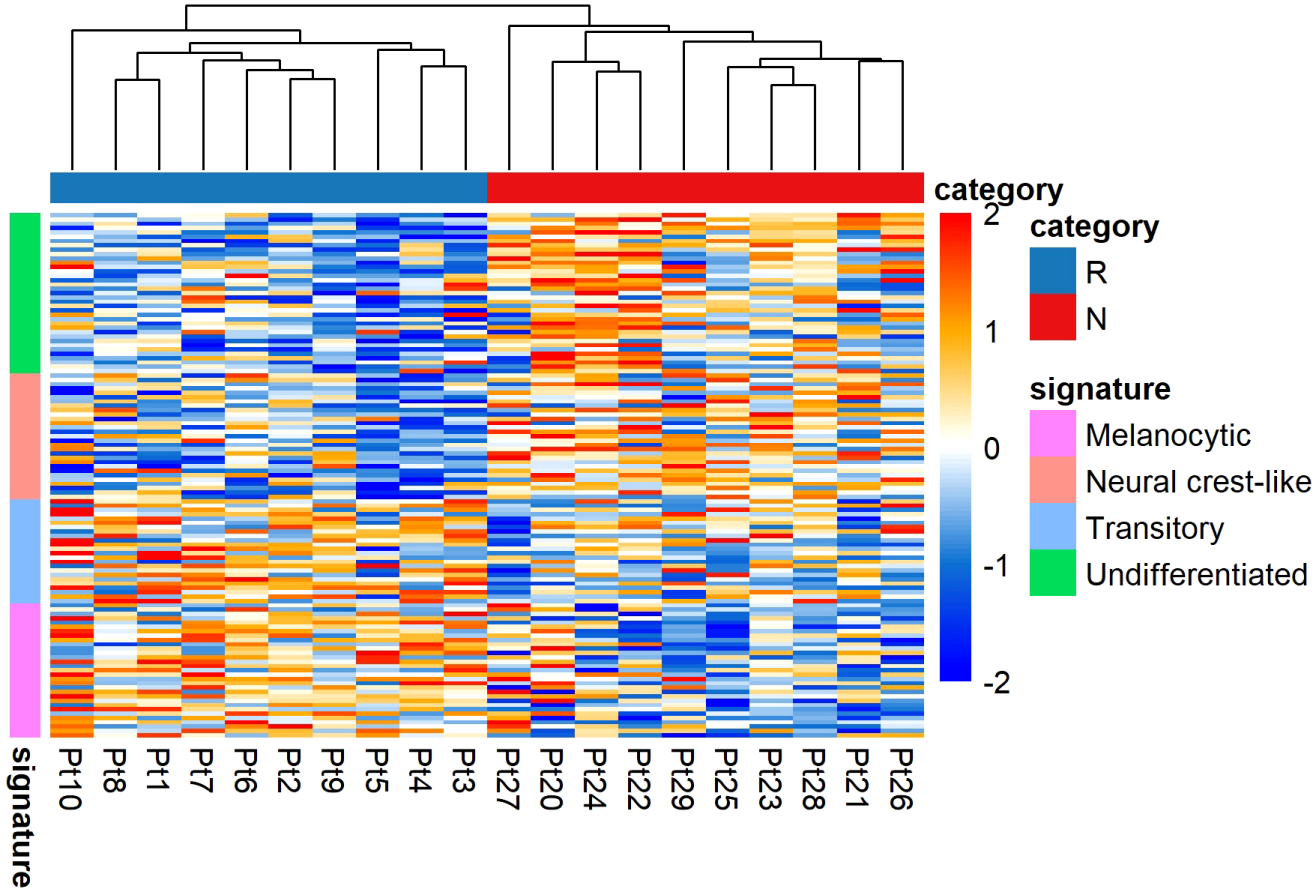
Transcriptomic states of responding (n = 10) versus non-responding (n = 10) melanomas. The non-responder group of melanomas exhibited undifferentiated and neural crest-like gene expression signatures. In contrast, the responder group of melanomas mainly exhibited melanocytic and transitory gene expression signatures. Values are in z-score. Here, Pt, patient; R, responder; N, non-responder.

To determine the altered transcriptomic state in the non-responding tumours compared to the responding tumours, we performed unsupervised hierarchical clustering using Tsoi et al. (41) gene sets (see **Supplementary Table S1**). Tsoi and colleagues identified that melanomas could exhibit four major transcriptomic states that are coupled to a differentiation trajectory corresponding to (1) undifferentiated, (2) neural crest-like, (3) transitory, and (4) melanocytic. Our data analysis confirmed that the non-responding tumours were enriched for genes characteristic of the undifferentiated and neural crest-like states. The responding tumours were mainly characterized by transitory and melanocytic gene signatures (**Figure 5**).

The presence of distinct phenotypic diversity was further confirmed in analyses with pre-treated melanoma samples, using bulk RNA-Seq datasets (GEO: GSE78220) and (GEO: GSE91061) (**Supplementary Figures S4A, B**). These data also showed that non-responding melanomas were enriched for genes characteristic of the undifferentiated and neural crest-like states.

In our previous study, Jeffs et al. (56) identified gene expression signatures that were associated with *MITF* high and low expression, which were observed in melanoma cell lines that were either non-invasive, or invasive, respectively (**Supplementary Figure S5B**). Independently, Widmer et al. (40) classified melanoma cell lines with *MITF* high and low expression as proliferative, and invasive, and identified respective signatures of gene expression in melanoma (**Supplementary Figure S5A**). We used these two independent gene expression signatures (40, 56) to distinguish invasive, non-invasive, and proliferative cell phenotypes from our transcriptomic data between responder and non-responder melanomas. In each analysis, we revealed that the non-responding melanomas were associated with an invasive gene signature (**Supplementary Figure S5**).

### Loss of interferon-gamma signalling, downregulation of cell apoptosis, and DNA damage repair pathways contribute to anti-PD1 resistance

**Figure 6:**
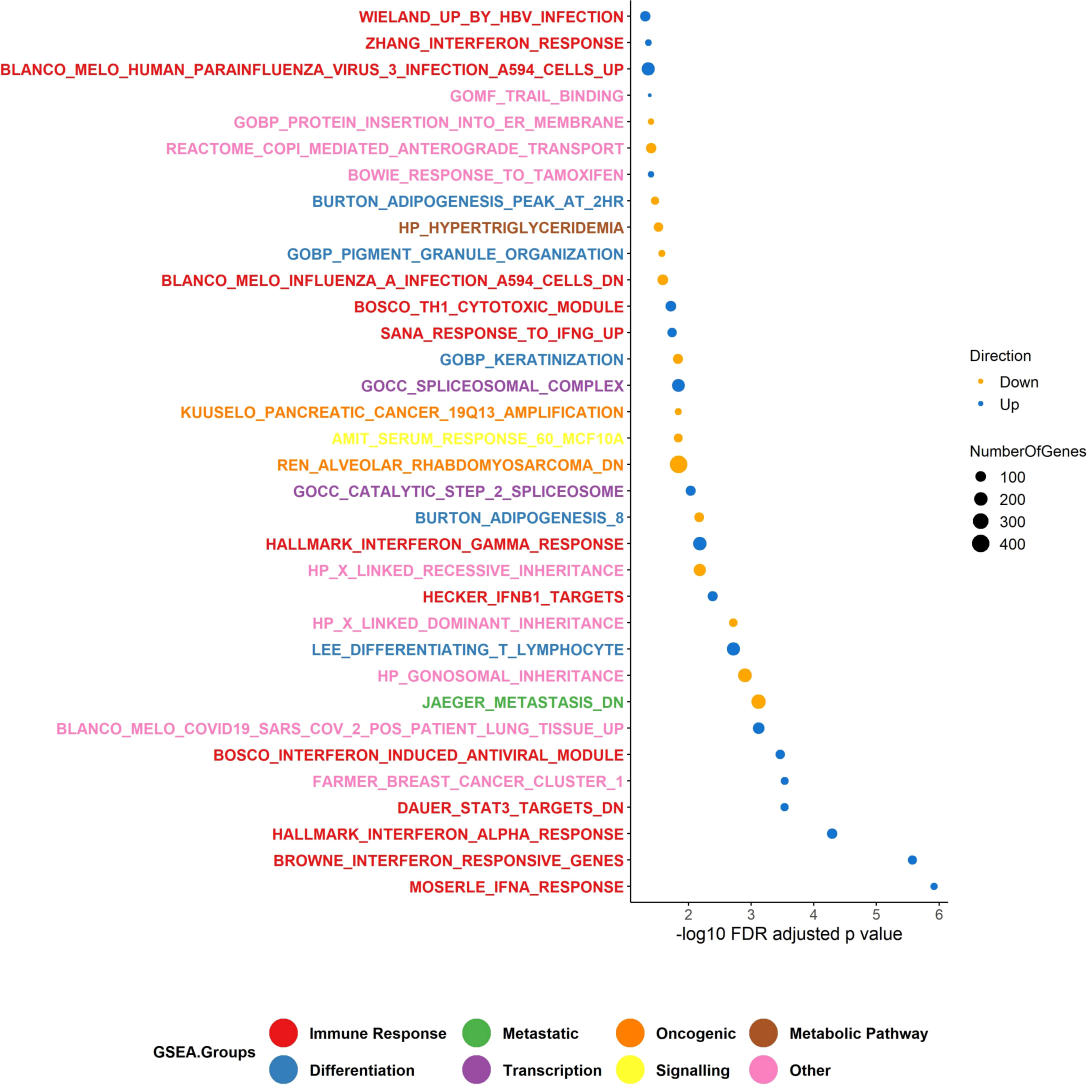
Gene set enrichment analysis (GSEA). The upregulated GSEA groups in the responder group of patients were enriched for genes involved in the immune response pathway and transcription, and the down-regulated genes were enriched for genes in differentiation, viral mimicry, and metabolic pathways. Number of responders = 10 and non-responders = 10. In total, 34 gene sets were identified in GSEA using CAMERA test. The p-value is <0.05.

We performed gene-set enrichment analysis using CAMERA (35) from our RNA-Seq data. From the gene-set enrichment analysis, thirty-four gene sets were found to be significantly altered (FDR adjusted p value threshold of 0.05). Of these, 198 genes involved in interferon gamma (IFN-γ), and 48 genes in Signal transducer and activator of transcription 3 (STAT3) signalling, were among the most significantly upregulated processes in the responding group (FDR adjusted p-value = 0.006 for HALLMARK_INTERFERON_GAMMA_RESPONSE, p-value = 0.0002 for DAUER_STAT3_TARGETS_DN) (**Figure 6**). This would be expected, given that the non-responding group of melanomas harboured relatively few immune cells. For the non-responding group of melanomas, approximately 243 genes were significantly involved in metastatic processes (FDR adjusted p-value = 0.0007 for JAEGER_METASTASIS_DN). Our data suggest that, IFN-γ in the responding melanomas might produce an anti-tumour effect by generating anti-proliferative and pro-apoptotic effects, enhancing increased tumour antigen presentation, and recruiting other immune cells (57).

**Figure 7:**
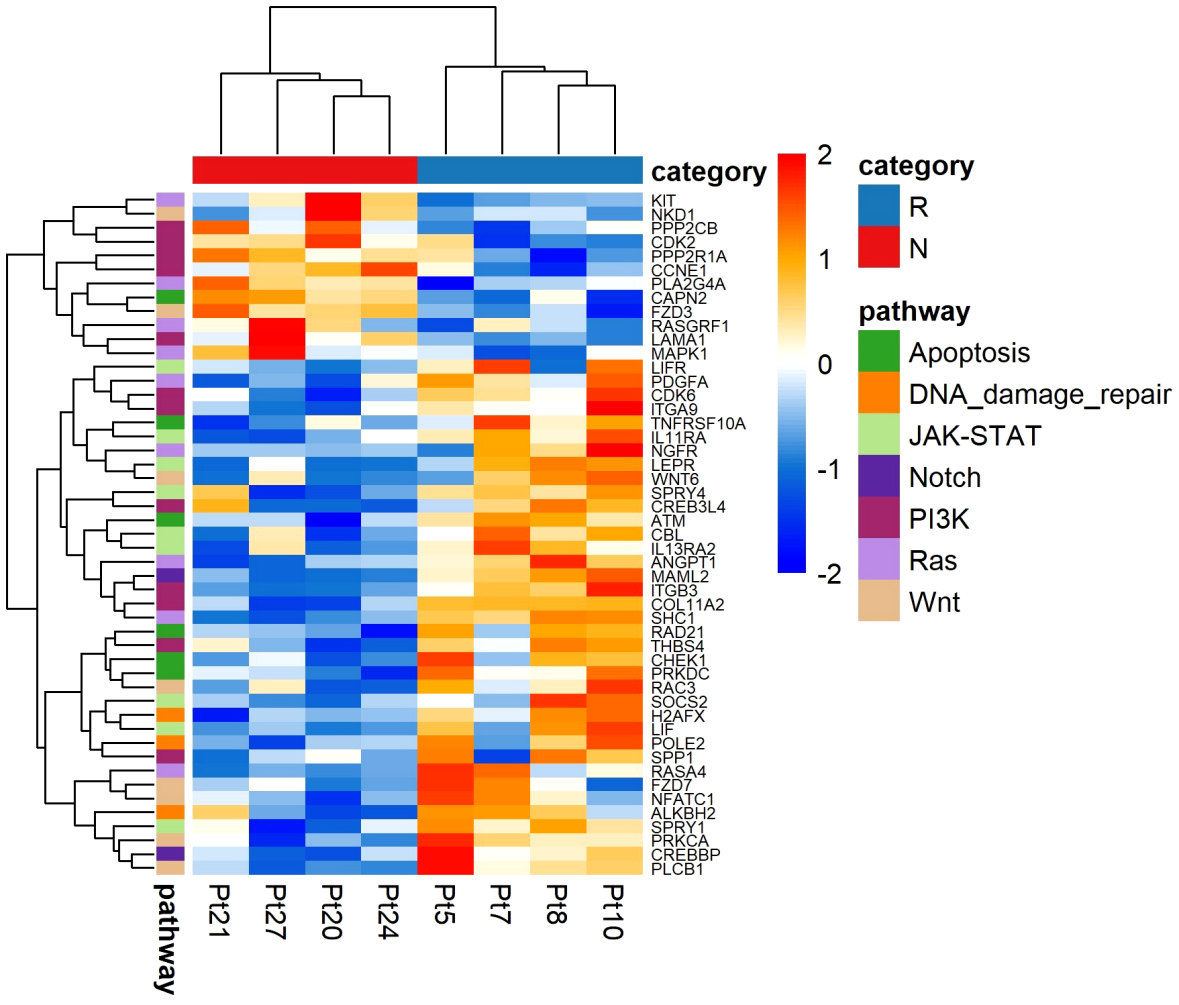
NanoString analysis of pathway-associated gene expression differences between responder (n = 4) and non-responder (n = 4) melanomas to anti-PD1 therapy. Values are in z-score. Here, Pt, patient; R, responder; N, non-responder.

To investigate which biological processes were altered in the non-responding melanomas compared to the responding melanomas, we performed pathway analysis using NanoString gene expression analysis, which was carried out using eight melanoma tissues (four responders and four non-responders), due to having limited amounts of RNA available. Interestingly, genes involved in cell apoptosis (*CAPN2*, *ATM*, *CHEK1*, *PRKDC*, *RAD21* and *TNFRSF10A*) and DNA damage repair (*ALKBH2*, *H2AFX*, *POLE2*) pathways were significantly upregulated in the responding melanomas, indicating that tumour intrinsic factors in responding melanomas were not as extremely altered as in non-responding tumours (**Figure 7**). In addition, our data suggest IFN-γ may trigger antiviral and adaptive immune responses through a Jak-STAT signalling pathway (**Figure 7**). Genes involved in these pathways were downregulated in the non-responder group of melanomas (**Table 2**), which may ultimately contribute to melanoma immune escape.

**Table 2:** Jak-Stat pathway genes and their functions.

Gene name	Fold change	p-value	Description
*CBL*	1.82	0.02	essential for T cell activation and regulating peripheral T cell tolerance (58)
*LEPR*	2.37	0.007	regulates both innate and adaptive responses through modulation of immune cells survival and proliferation as well as increasing the cytotoxicity of NK cells, and activating granulocytes, macrophages and DCs (59)
*SOCS2*	1.733	0.02	involved in regulation of developmental and homeostatic pathways and loss of *SOCS2* are involved in *STAT3* de-activation (60)
*SPRY1*	2.58	0.02	inhibits signalling from various growth factors pathways and play role as a tumour suppressor in various malignancies (61)
*SPRY4*	3.11	0.03	involved in cell proliferation and lower expression of *SPRY4* predicts poor prognosis (62)

Overall, this analysis hinted that multiple tumour intrinsic factors such as loss of interferon-gamma (IFN-γ) signalling pathways, lack of T cell responses due to loss of tumour antigen expression, downregulation of cell apoptosis pathway and DNA damage repair may have contributed to immunotherapy resistance.

### Non-responding melanomas have an increased expression of genes involved in epithelial mesenchymal transition and viral mimicry

**Figure 8:**
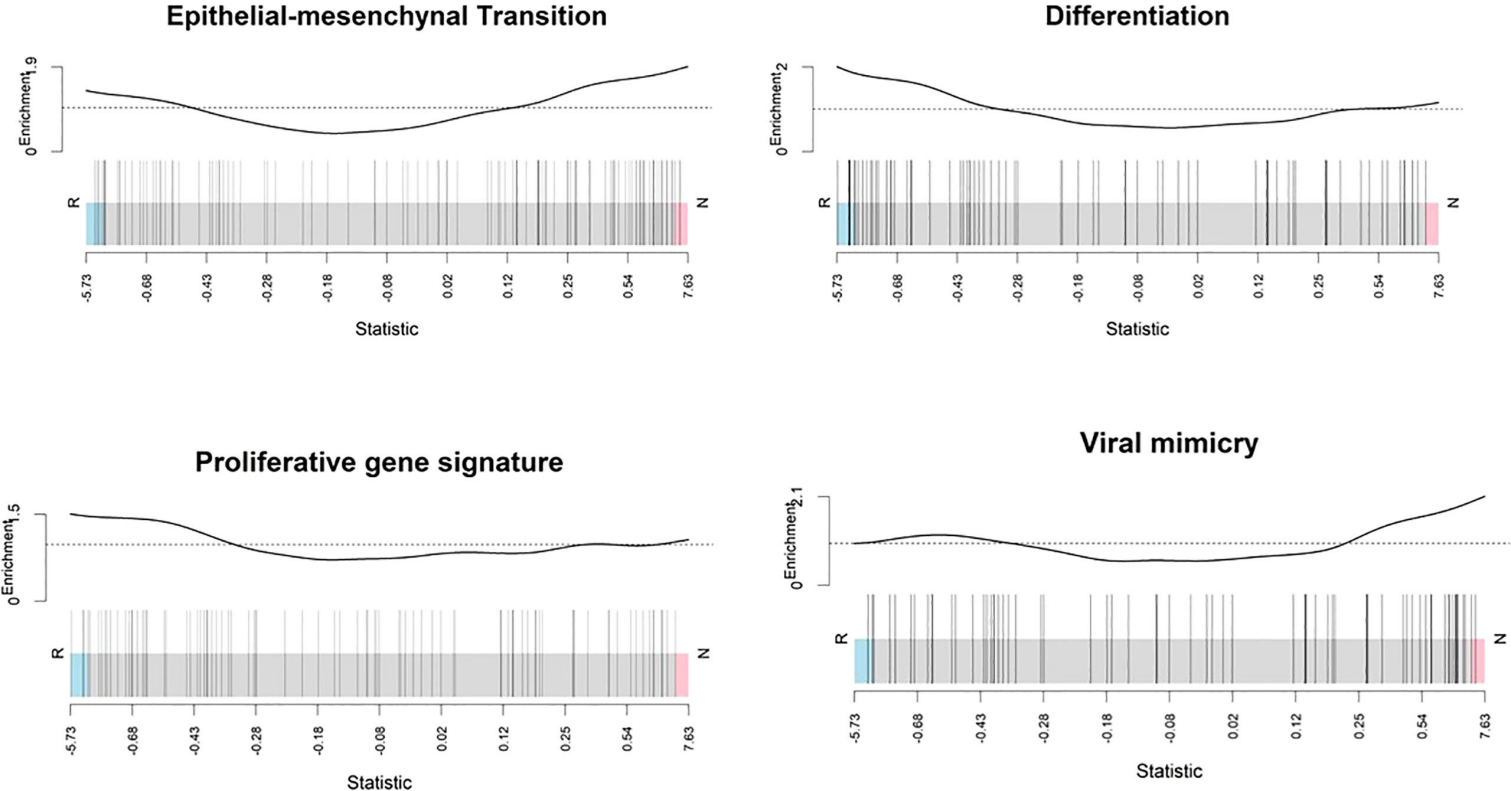
Enrichment score analysis. Barcode plots show that genes involved in the epithelial mesenchymal transition and viral mimicry detection were upregulated in non-responders whereas differentiation-associated genes and proliferative signature genes were downregulated. Genes expressed in the profile datasets were ranked by log2 fold changes (high-expression/low-expression) in the responders (left side, n = 10) and non-responders (right side, n = 10). Overall shifts in the genes (represented by vertical bars) towards the left or right give an indication of the overall gene level for specific pathways rather than at the individual gene level. Above each barcode plot enrichment scores are shown. Positive and negative values for enrichment scores mean positive and negative enrichment, respectively. Here, R, responder; N, non-responder.

In non-responder melanomas there was enrichment of genes involved in epithelial mesenchymal transition, and viral mimicry pathways, while in responder melanomas differentiation genes were enriched (**Figure 8**). EMT signature genes included *CDH1*, *EPCAM*, *GRHL2*, *KRT19*, *RAB25*, *CDH2*, *VIM*, *ZEB1*, *ZEB2*, *SNAI2* and *TWIST1*, collected from Tan and colleagues (39). Viral mimicry pathway genes were obtained from our previously published work (38), and included 12 genes, which play roles ranging from pattern recognition receptors that detect dsRNA and dsDNA (*DDX58*, *DDX41*, *IFIH1*), to activation of mitochondrial antiviral signalling proteins (*MAVS*), and transcription factors (*IRF7*, *IRF1*) and the activation of interferons (*IFI27*, *IFI44*, *IFI44L*, *IFI16*). However, overexpression of *DDX58* is responsible for local immunosuppression in the tumour bed and is associated with poor prognosis and higher tumour grade of ovarian cancer (63). Differentiation gene set and proliferative signature gene plots showed that responding melanomas were enriched with a gene set associated with a proliferative state or melanocytic stages of melanoma differentiation. In contrast, the non-responding melanomas were enriched with invasiveness genes.

## Discussion

In this study we have focused on systematic assessment of responder and non-responder melanoma patients to ICI treatment with respect to TMB, expressed neoantigens, deconvolution analysis of the tumour immune microenvironment, and phenotype switching, through using Oncomine Next Generation Sequencing and RNA-Seq analysis, which were generated from melanoma tissues. In all cases the melanoma tissues were obtained prior to the patients receiving anti-PD1 therapy. We found that, as others have previously reported, when compared to non-responding melanomas, the responding melanomas were more likely to have accumulated a greater tumour mutational burden.

Tumour mutational burden (TMB) refers to the total number of non-synonymous mutations observed per megabase (64), and it is a genomic biomarker with relatively low power to predict favourable responses to ICIs in melanoma (65). High TMB results in the expression of tumour related antigens, or neoantigens, that can trigger T cell activation. We observed that TMB had a strong positive correlation with expressed neoantigens, and a moderate positive relationship with immune scores in melanoma patients.

A possible reason that melanomas frequently don’t respond to immune checkpoint therapy could be due to lack of recognition of tumour cells by T cells, which may be linked to an absence of tumour neoantigens (66). Clinical responses to ICIs are thought to be mediated in part by T cell recognition of neoantigens that are derived from cancer-specific mutations and presented on major histocompatibility complex (MHC) molecules (67). Moreover, our results showed that non-responding tumours were highly enriched with malignant cell types, CAF cell subsets, an M2 subtype of macrophages, and B cells, compared to the responding tumours. A higher abundance of CAFs in the tumour stroma is associated with an increased risk of invasion, metastasis, and poor prognosis of melanoma through the release of a variety of chemokines and cytokines, extracellular matrix (ECM) components and ECM-remodelling enzymes (51). Previously, several studies have reported that melanoma-associated fibroblasts can induce immune suppression *via* melanoma–stroma crosstalk (51–53). We therefore carried out a deconvolution analysis of melanoma RNA-Seq data to identify immune cell components, and we also investigated gene expression patterns characteristic of phenotype switching.

Melanocytic differentiation pathways are thought to have a role in controlling cell migration of the neural-crest lineage. One of the most established factors that controls neural-crest migration, and melanocyte differentiation, is the expression level of microphthalmia-associated transcription factor (*MITF*). In melanoma cell biology, *MITF* is a central player, controlling aspects of phenotypic switching (68), and is one of several critical transcriptional regulators involved in melanocyte development, upregulating a set of genes to drive melanocytic differentiation (3). All melanomas exhibit at least two distinct *MITF* transcriptional cell states, which are represented by melanomas with high levels of *MITF*, versus melanomas with low levels of *MITF* (50). High *MITF* levels are thought to promote cell proliferation through the direct activation of the *DIAPH1* gene, and low *MITF* expression in melanoma is associated with cell migration (68). We therefore used gene expression signatures identified by Jeffs et al. (56), and characterised by high and low *MITF* expression levels, to distinguish invasive and non-invasive cell phenotypes between responding and non-responding tumour types. In addition, we characterised our dataset using a related, but independent gene signature matrix associated with high and low *MITF* expression levels, as identified by Widmer et al. (40). In each scenario, using our dataset, non-responding melanomas were associated with an invasive gene signature, while responding melanomas were associated with proliferative and non-invasive gene signature matrices.

The acquisition of differentiation plasticity is a non-mutational mechanism of drug resistance in melanoma. This was also part of our rationale for investigating an association with anti-PD1 treatment resistance, using gene signatures associated with a melanoma differentiation trajectory, as categorized by Tsoi et al. (41). Our results confirmed that non-responding melanomas were enriched for genes that were characteristic of undifferentiated and neural crest-like differentiation states, while in contrast, the responding melanomas mainly exhibited expression signatures characterized by transitory and melanocytic state gene signatures. To further validate these findings, we next analysed other publicly available ICI-associated melanoma RNA-Seq data (6, 8), in which we observed very similar outcomes, which further supports the notion that the responding versus non-responding melanomas exhibit phenotype switching behaviour.

**Figure 9:**
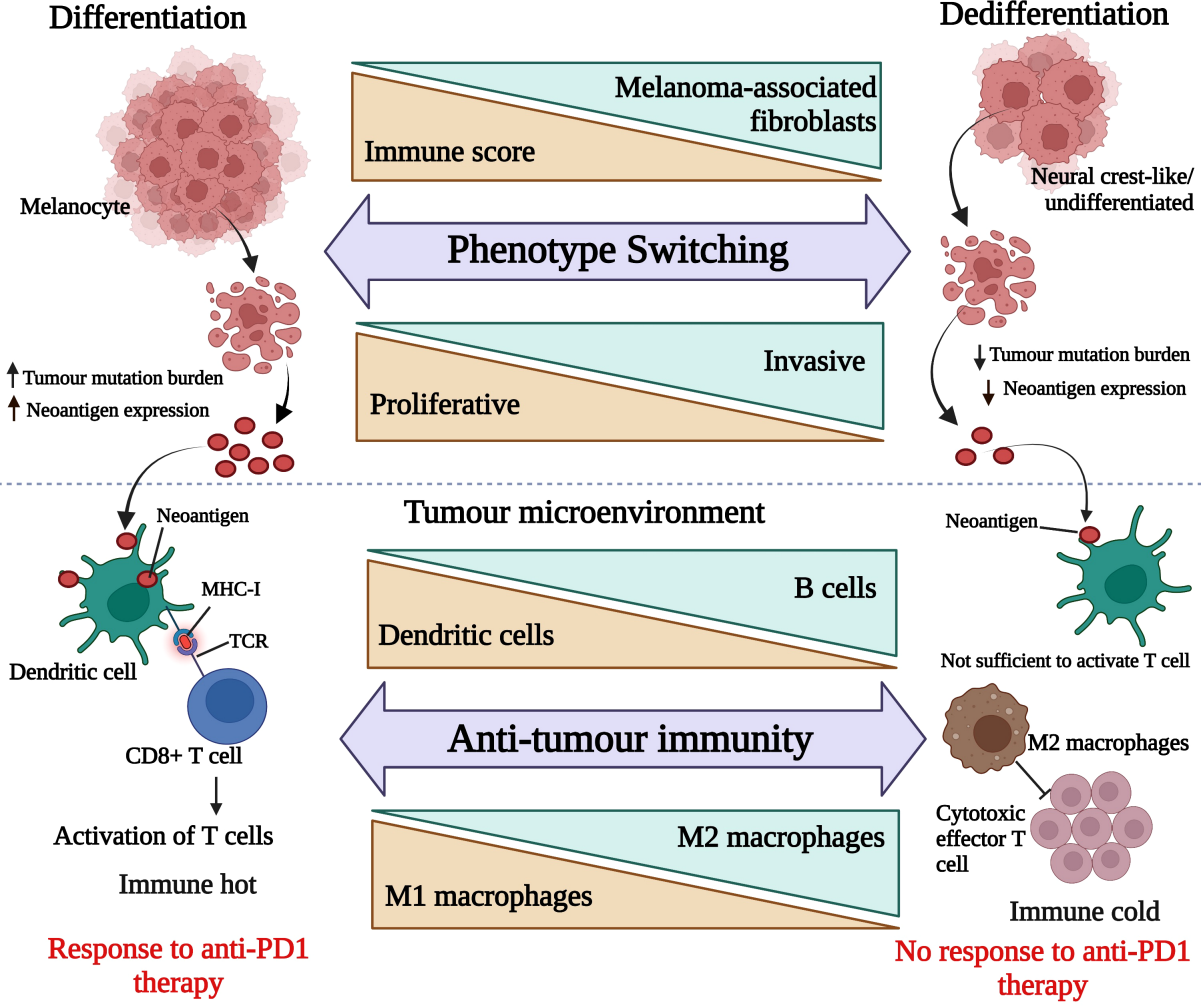
Hypothetical presentation of responding tumour microenvironment versus non-responding tumour microenvironment. The upper panel is representing melanoma de-differentiation trajectory and the lower panel is focusing on the interplay between melanoma cell types and immune cell composition. This image was generated using BioRender.com.

From our findings, we propose a model (see **Figure 9**), which puts forward the idea that responding melanomas are mainly melanocytic in nature and due to their proliferative nature, they frequently (but not always) generate relatively larger tumour mutation burdens leading to higher neoantigen production. In contrast, non-responding melanomas are mainly composed of cells exhibiting neural crest-like or undifferentiated transcriptomic stages, which makes them more neural crest-like/cancer stem cell-like in nature and less proliferative. Due to their lower rate of proliferation, they produce a relatively smaller mutation burden, resulting in less neoantigen expression.

From our RNA-Seq data we found that responding melanomas were mostly expressing IFN-γ, STAT3, cell apoptosis and DNA damage repair signalling pathway genes, whereas non-responding melanomas significantly expressed metastatic pathway genes, representing important differences in cell signalling. Our data also suggested that IFN-γ in the responding tumours triggered antiviral and adaptive immune responses through the Jak-STAT signalling pathway (**Figure 7**) – Among the genes involved in these pathways were *CBL*, *LEPR*, *SOCS2*, *SPRY1*, and *SPRY4*, which were downregulated in non-responder melanomas (**Table 2**) and ultimately contributed to melanoma immune escape. Interestingly, IFN-γ plays a dual role in anti-tumour immune response – firstly, through anti-tumour immune responses, directing anti-proliferative and pro-apoptotic effects on tumour cells, enhancing tumour neoantigen presentation, and immune cell recruitment (57). Secondly, IFN-γ can cause immune escape by increasing the expression of PD-L1 on the surface of tumour cells (48).

IFN-γ is mainly produced by Th1 cells, and is critical for immune responses and sustained M1 macrophage bioactivities to eliminate neoplastic cells (69). Supporting this, responder melanomas were highly enriched with a combination of CD8+ T cells, T helper cells (Th1 and Th2), dendritic cells (DCs) and an M1 subtype of macrophages, whereas non-responding tumours contained abundant M2 macrophages and B cells. Th1 cells, IFN-γ, and TNFα regulate M1 macrophages to enhance antigen presentation on the major histocompatibility complex (MHC) (70, 71) to exert anti-tumour immune response. In contrast, activated M2 macrophage subtypes possess pro-tumorigenic properties (72, 73). Increased levels of Th1 and Th2 cells in responding tumours activate CD8+ T cells through IFN-γ as a mediator, which inhibits tumour cell proliferation. Th1 cells in responding tumours activate macrophages and maturation of DCs (54), which then recognize tumour antigens, and process neoantigens. DCs also induce a cross-presentation of antigens to CD8+ T lymphocytes by MHC class I molecules, and to CD4+ by MHC class II molecules to initiate and regulate both innate and adaptive immunity. Furthermore, mature DCs can contribute to the cytotoxic immune reaction directly, as well as activating NK cells (54), which are also linked with favourable clinical outcomes in melanoma. Additionally, our study showed that non-responding melanomas were enriched with B-cells (**Figure 4**), and they exhibited higher expression of chemokine C-X-C motif ligand 13 (*CXCL13*) (p value = 0.02, Mann-Whitney U), compared to responding melanomas (**Supplementary Figure S6**). Interestingly, *CXCL13* expression facilitates recruitment of B cells, and leads to an immune-suppressive TME, promoting initiation of tumorigenesis, tumour progression and metastasis (74, 75). In addition, several studies have reported that CXCL13 modulates cancer stem cell properties by recruiting B-cells (74, 76). Overall, these reports are consistent with our data.

Therefore, as depicted in **Figure 9**, we surmise that the responding melanoma bed was composed of larger numbers of activated DCs, capturing the neoantigens and presenting them on MHC molecules to T cells, resulting in their activation, which is reflective of an immunologically “hot” tumour microenvironment. On the other hand, non-responding melanomas lacked activated DCs, and produced fewer neoantigens, which led to insufficient signals to trigger activation of T cells. In addition, the abundance of M2 macrophages in the non-responding melanoma bed inhibited effector T cell activation. This together with de-differentiated gene expression signatures, was associated with innate resistance to anti-PD1 therapy, and led to an immunologically “cold” tumour environment for melanomas.

Our study expands knowledge of the TMB, expressed neoantigens, tumour immune microenvironment and phenotype switching in melanoma, although our study had certain limitations. Due to the retrospective nature of the study, the responding and non-responding melanomas to anti-PD1 therapy represented relatively small numbers of melanomas, and the relative numbers of melanomas in each group were not representative of the relative incidence in the entire malignant cutaneous melanoma population. Additionally, the conclusions are drawn from bioinformatic analyses of an initial sample cohort from a single institution. Further validation using *in vivo* malignant melanoma models, as well as larger cohort studies are thus warranted.

## Conclusion

Our results suggest that TMB positively correlated with neoantigen expression in melanoma, and TMB also exhibited a moderate positive relationship with melanoma immune scores. Additionally, we identified that responding melanomas were comprised mainly of tumour cells with relatively more highly differentiated, and proliferative gene expression signatures, which were associated with comparatively immunologically “hot” tumour microenvironments. Furthermore, our data suggest that immunogenicity in the melanoma tumour microenvironment was associated with the interplay between the transcriptomic state(s) of the tumour cells, neoantigen expression and immune cell types present in the tumour bed, highlighting the need for alternative therapeutic strategies to target dormant cell types in non-responding melanomas.

## Data availability statement

The original contributions presented in the study are publicly available. This data can be found here: The accession number for the RNA-Seq transcriptome data is GEO: GSE213145.

## Ethics statement

This study was reviewed and approved by Krakow Branch of the Maria Sklodowska-Curie National Research Institute of Oncology (KB/430-74/20). The patients/participants provided their written informed consent to participate in this study.

## Author contributions

ME, AC, and SH: conceptualization. SH and GG: data curation. SH, GG, PS, PT, and CP: formal analysis. ME, AC, and CJ: funding acquisition. SH: writing—original draft. ME, SH, GG, PS, PT, CP, AC, SA, and MR: writing—review and editing. CJ, JR, BC-S, MR and AH-L: sample collection. All authors contributed to the article and approved the submitted version.

## Funding

This work was supported by project grant funding from the Health Research Council of New Zealand, grant number 18/144, Royal Society of New Zealand Marsden Fund, grant number 16-UOO-178, grant funding from Otago Medical Research Foundation, and from the Maurice Wilkins Centre for Molecular Biodiscovery, a University of Otago PhD Scholarship (SH), and a Rutherford Discovery Fellowship (AC).

## Conflict of interest

The authors declare that the research was conducted in the absence of any commercial or financial relationships that could be construed as a potential conflict of interest.

## Publisher’s note

All claims expressed in this article are solely those of the authors and do not necessarily represent those of their affiliated organizations, or those of the publisher, the editors and the reviewers. Any product that may be evaluated in this article, or claim that may be made by its manufacturer, is not guaranteed or endorsed by the publisher.
