# Supplementary material for: Innate immune checkpoint inhibitor resistance is associated with melanoma sub-types exhibiting invasive and de-differentiated gene expression signatures
Source: Front Immunol. 2022 Sep 28;13:955063. doi: 10.3389/fimmu.2022.955063 (PMC9554309; doi:10.3389/fimmu.2022.955063)
Supplement: Supplementary file 1 [file DataSheet_1.pdf]

Supplementary Material.

Hossain et al., Innate immune checkpoint inhibitor resistance is associated with melanoma sub-types exhibiting invasive and de-differentiated gene expression signatures

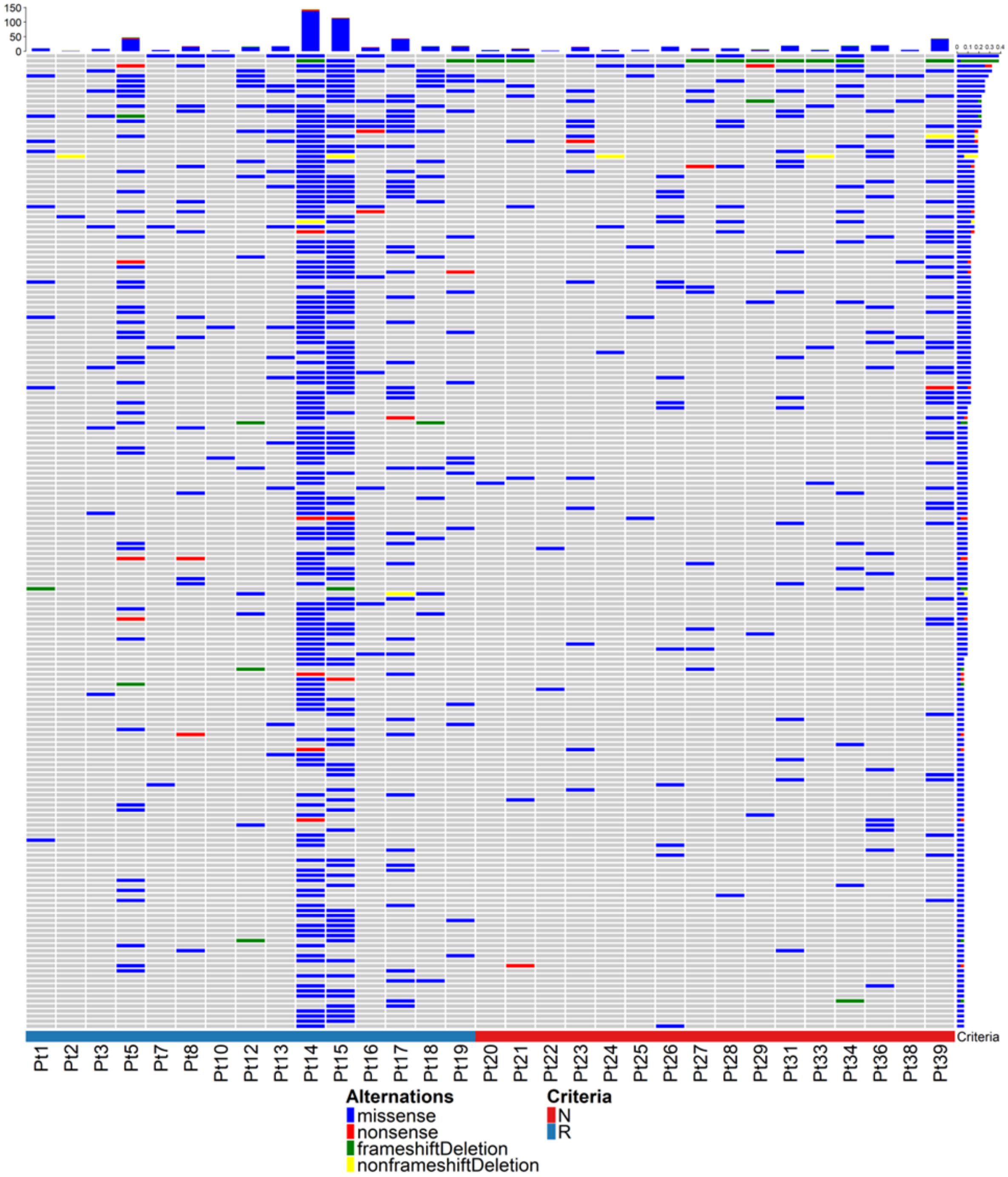

Supplementary Figure S1: The total number of nsSNVs (non-synonymous single nucleotide variants) detected in melanomas. Top, the total nsSNV load of each melanoma tumour. The heatmap represents only 31 samples because these samples passed an in-built QC (quality

control) test with ≥80 % depth of read uniformity in sequencing using the Oncomine Tumour Mutation Load – w3.0 – DNA – Single Sample in the Ion Reporter cloud server.

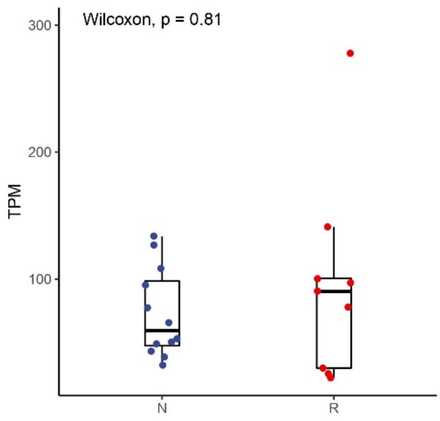

Supplementary Figure S2: HLA expression level in responder (n = 9) and non-responder (n = 11) tumours in TPM (transcripts per million) (p = 0.81, not significant, Wilcoxon signed-rank test). On the horizontal axis, R = responder, N = non-responder.

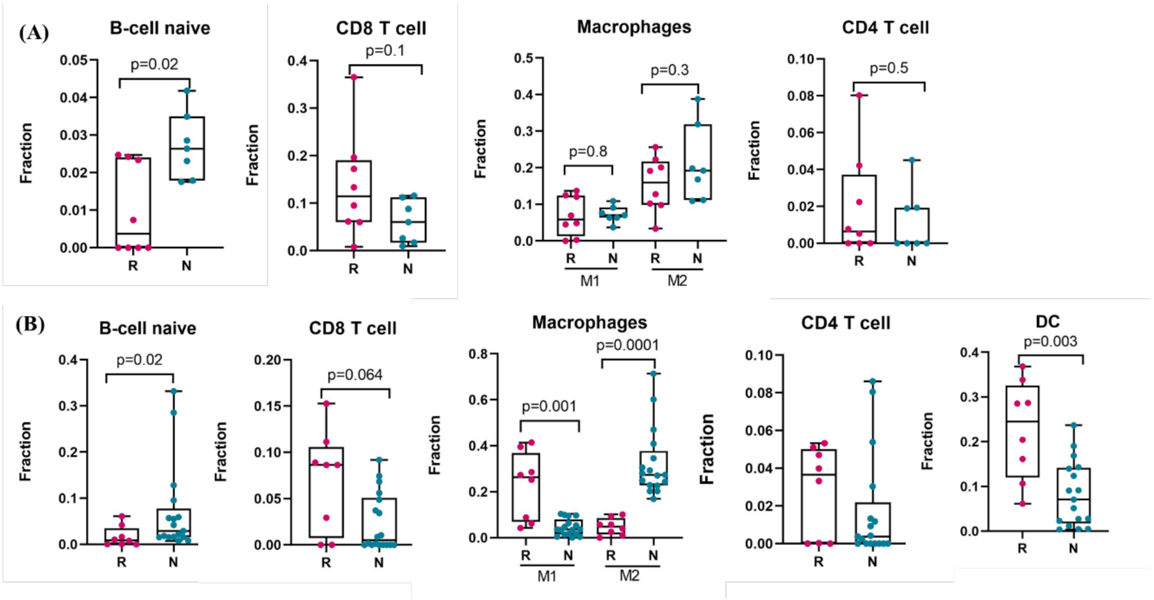

Supplementary Figure S3: Deconvolution study to estimate different immune cell abundance in responding and non-responding melanoma tissues using online available datasets. **(A)** Immune cell proportions between responder (n = 8) and non-responder (n = 7) melanomas using the Hugo *et al* (1) dataset. **(B)** Immune cell proportions between responder (n = 8) and non-responder (n = 17) melanomas using the Riaz *et al* (2) dataset. The significance test was performed using Mann–Whitney U test. On the horizontal axis, R = responder, N = non-responder. DC = dendritic cells.

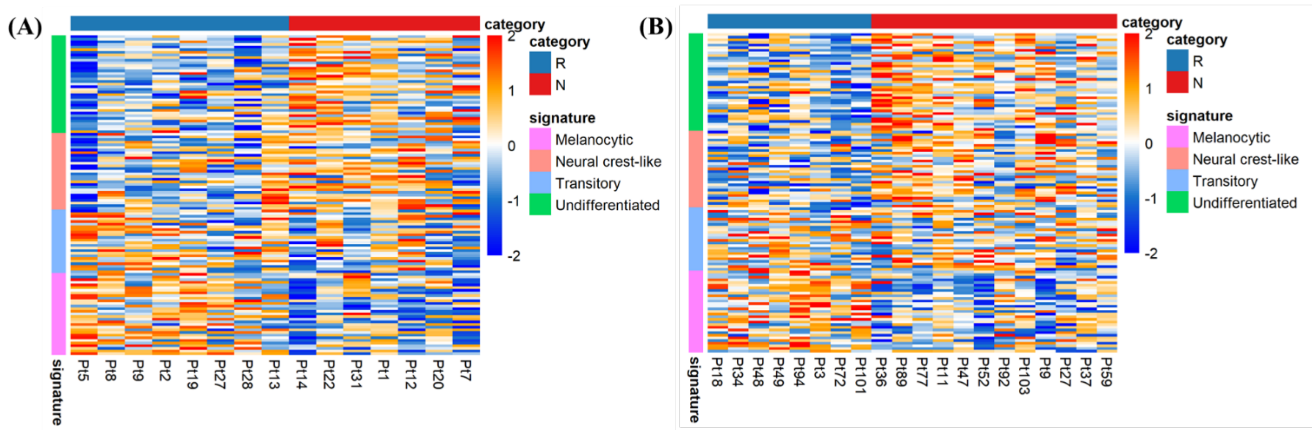

Supplementary Figure S4: Transcriptomic states of responding versus non-responding melanomas using online available datasets. **(A)** Heatmap showing the expression level of phenotypic genes between responder (n = 8) and non-responder (n = 7) melanomas using the Hugo *et al* (1) dataset. **(B)** Heatmap showing the expression level of phenotypic genes between responder (n = 8) and non-responder (n = 12) melanomas using the Riaz *et al* (2) dataset. Values are in z-score. Here, Pt = patient, R = responder, N = non-responder.

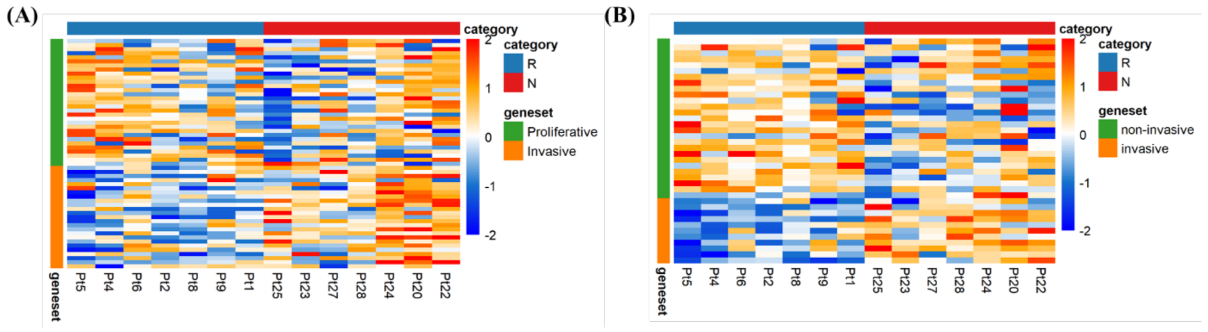

Supplementary Figure S5: A non-responder group of melanomas exhibited higher expression of an invasive gene signature compared to the responder group of melanomas. **(A)** Heatmap showing the expression level of invasive and proliferative genes between responder (n = 7) and non-responder (n = 7) melanomas. Hierarchical clustering is shown for the samples and genes. **(B)** Heatmap showing the expression level of invasive (n = 7) and non-invasive (n = 7) genes between responder and non-responder melanomas. The melanoma invasive gene signatures were derived from 2 independent studies (Jeffs and Widmer – (3, 4)). Values are in z-score. Here, Pt = patient, R = responder, N = non-responder.

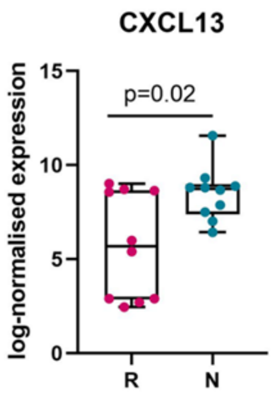

Supplementary Figure S6: Expression of *CXCL13* between responder (n = 10) and non-responder (n = 10) melanomas. The significance test was performed using Mann–Whitney U test. On the horizontal axis, R = responder, N = non-responder.

Supplementary Table S1: Signature genes for each phenotype were acquired from Tsoi and colleagues (5). These gene sets were used to prepare Figure 5.

Gene name	Phenotype
ZNF467	Undifferentiated
ZNF185	Undifferentiated
TNFAIP2	Undifferentiated
TMEM200A	Undifferentiated
TMEM184A	Undifferentiated
TRERF1	Undifferentiated
SAMD11	Undifferentiated
SOX9	Undifferentiated
SLC16A14	Undifferentiated
SERPINB7	Undifferentiated
PSG4	Undifferentiated
PBX1	Undifferentiated
PLAGL1	Undifferentiated
PHLDB2	Undifferentiated
PLEKHA6	Undifferentiated
NUAK1	Undifferentiated

MECOM	Undifferentiated
LYPD6B	Undifferentiated
LAMA5	Undifferentiated
IL7R	Undifferentiated
IL4R	Undifferentiated
GATA2	Undifferentiated
GPRC5A	Undifferentiated
FLNC	Undifferentiated
FERMT1	Undifferentiated
FAT4	Undifferentiated
EGFR	Undifferentiated
CRIM1	Undifferentiated
CDK15	Undifferentiated
CCDC69	Undifferentiated
F2RL1	Undifferentiated
CARD11	Undifferentiated
CREB3L1	Undifferentiated
CNN1	Undifferentiated
BNC1	Undifferentiated
ATP8B1	Undifferentiated
AR	Undifferentiated
CXCL8	Neural crest-like
CEMIP	Neural crest-like
WNT5A	Neural crest-like
TGFBI	Neural crest-like
TGFA	Neural crest-like
TFAP2C	Neural crest-like
TSPAN13	Neural crest-like
SOX2	Neural crest-like
SLC24A3	Neural crest-like
SLITRK6	Neural crest-like
SHISA2	Neural crest-like

SEMA3B	Neural crest-like
RAMP1	Neural crest-like
PMEPA1	Neural crest-like
PLA2G7	Neural crest-like
NTM	Neural crest-like
MUC5B	Neural crest-like
LRRC15	Neural crest-like
ITGB8	Neural crest-like
GLI2	Neural crest-like
FOXC2	Neural crest-like
FLT1	Neural crest-like
CYB5R2	Neural crest-like
CSRP2	Neural crest-like
CCL2	Neural crest-like
CADM3	Neural crest-like
CADM1	Neural crest-like
CD96	Neural crest-like
AIM2	Neural crest-like
XYLT1	Transitory
TSPAN7	Transitory
SOD3	Transitory
SCRG1	Transitory
SELENBP1	Transitory
RNASE1	Transitory
RAPGEF4	Transitory
PCDH7	Transitory
PRSS33	Transitory
PCSK6	Transitory
PLBD1	Transitory
NPR1	Transitory
MMP15	Transitory
MAMDC2	Transitory

LSAMP	Transitory
LRRTM4	Transitory
GDF11	Transitory
FXYD3	Transitory
EBF3	Transitory
COL11A2	Transitory
COL9A1	Transitory
CX3CL1	Transitory
ANO4	Transitory
ALDH1A1	Transitory
CCDC171	Melanocytic
CFAP61	Melanocytic
ZDHHC11B	Melanocytic
TNFRSF14	Melanocytic
TPPP	Melanocytic
TRIM63	Melanocytic
TRPM1	Melanocytic
TSPAN10	Melanocytic
SLC7A8	Melanocytic
SEMA6A	Melanocytic
RNF144B	Melanocytic
RGS12	Melanocytic
NR4A3	Melanocytic
NAV2	Melanocytic
MYO1D	Melanocytic
MAPK4	Melanocytic
MLANA	Melanocytic
KCP	Melanocytic
IL16	Melanocytic
IL12RB2	Melanocytic
GOLGA7B	Melanocytic
GPR143	Melanocytic

EPHA5	Melanocytic
C2orf88	Melanocytic
CEACAM1	Melanocytic
CAPG	Melanocytic
CDH3	Melanocytic
ATP6V0D2	Melanocytic
ABCD1	Melanocytic
ABCB5	Melanocytic
ADAM23	Melanocytic

Supplementary Text for Table 1:

Among the 19 responders, most were above 60 years old (13 patients). The median age of the total cohort was 65 years, 2 patients were aged below 40, 11 patients were aged between 40 – 60 years and 27 patients were above 60 years old. Of these melanomas, 9 out of 40 melanomas (~23%) were $BRAF^{V600E}$-mutation positive, with one of them aged below 40, five between 40 – 60 years and three were more than 60 years old. Within the nine $BRAF^{V600E}$ mutation positive patients, six were male and the remainder were female. A total of 25 out of 40 melanomas (62.5%) patients had lymph node positive metastasis, and among them eight had $BRAF^{V600E}$-positive mutations, while thirteen responded to the anti-PD1 therapy.

Reference:

1. Hugo W, Zaretsky JM, Sun L, Song C, Moreno BH, Hu-Lieskovan S, et al. Genomic and Transcriptomic Features of Response to Anti-PD-1 Therapy in Metastatic Melanoma. Cell. 2017;168(3):542.
2. Riaz N, Havel JJ, Makarov V, Desrichard A, Urba WJ, Sims JS, et al. Tumor and Microenvironment Evolution during Immunotherapy with Nivolumab. Cell. 2017;171(4):934-49.e16.
3. Jeffs AR, Glover AC, Slobbe LJ, Wang L, He S, Hazlett JA, et al. A gene expression signature of invasive potential in metastatic melanoma cells. PloS one. 2009;4(12):e8461.
4. Widmer DS, Cheng PF, Eichhoff OM, Belloni BC, Zipser MC, Schlegel NC, et al. Systematic classification of melanoma cells by phenotype-specific gene expression mapping. Pigment cell & melanoma research. 2012;25(3):343-53.
5. Tsoi J, Robert L, Paraiso K, Galvan C, Sheu KM, Lay J, et al. Multi-stage Differentiation Defines Melanoma Subtypes with Differential Vulnerability to Drug-Induced Iron-Dependent Oxidative Stress. Cancer cell. 2018;33(5):890-904.e5.
